# Perception of illusory contours in children and adults: An eye-tracking study

**DOI:** 10.3758/s13414-023-02832-z

**Published:** 2023-12-29

**Authors:** Michael Kavšek

**Affiliations:** Department of Psychology, University of Bonn, Kaiser-Karl-Ring 9, 53111 Bonn, Germany

**Keywords:** Subjective contour perception, Illusory contour perception, Development of global versus local processing, Eye tracking, Kanizsa illusion, Ehrenstein illusion

## Abstract

The eye-tracking study investigated the perception of subjective Kanizsa and Ehrenstein figures in adults and in children aged 3–4, 5–6, 7–8, and 9–11 years of age. More specifically, the distribution of looking at the inner stimulus part versus the inducing elements was measured for illusory figures, figures with real contours, and control displays. It was hypothesized that longer looking at the inner area of the illusory figures indicates global contour interpolation, whereas longer looking at the inducing elements indicates a local processing mode. According to the results, participants of all ages looked longer at the illusory Kanizsa and Ehrenstein contours than at the figures’ inducing elements. However, performance was lowest in the children aged 3–4 years and increased during the preschool period. Moreover, the illusory contour displays elicited comparable visual responses as did the real contour displays. The use of the control displays that contained no contour information ensured that the participants’ looking behavior was not driven by a spontaneous tendency to attend to the inner stimulus parts. The study confirms the view that sensitivity to illusory contours emerges very early in life.

## Introduction

**Fig. 1:**
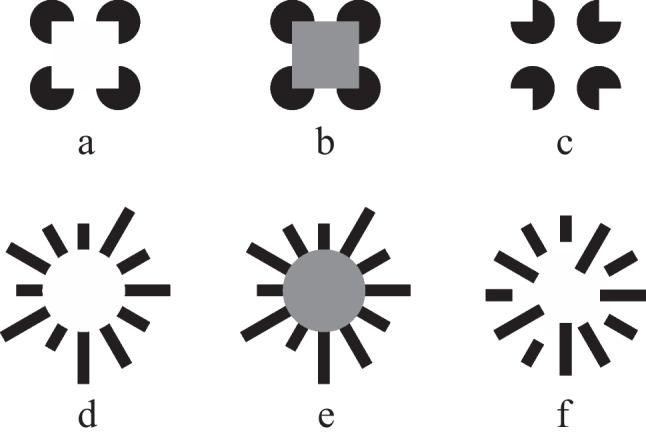
The stimuli employed in the study consisted of three versions of the Kanizsa and of three versions of the Ehrenstein figure: an illusory display (**a** and **d**), a real display (**b** and **e**), and a control display (**c** and **f**)

Subjective/illusory contours are contours that are perceived as complete shapes, although parts of the contours’ edges are not physically specified. Powerful examples are depicted in Fig. 1a, the subjective Kanizsa square (Kanizsa, 1976), and Fig. 1d, the subjective Ehrenstein circle (Ehrenstein, 1941). In the subjective Kanizsa figure, an illusory square is induced by four three-quarter circles. In the subjective Ehrenstein figure, an illusory circle is induced by the inner tips of converging lines.

Subjective contours are a tool for examining the way shapes are processed. For instance, the Kanizsa display in Fig. 1a might be perceived as a mere collection of four circles with cut-outs similar to the stimulus shown in Fig. 1c. If so, the observer would concentrate on the circles and neglect the inner area of the stimulus, that is, the observer would display a local processing mode. Alternatively, the contour information provided by the symmetrically arranged cut-outs of the circles in Fig. 1a might be combined into a coherent (illusory) square shape similar to the grey area in Fig. 1b. If so, the observer would attend to the inner area of the stimulus, that is, he would display a global processing mode. In general, attention to the inducers, that is, the disconnected, visible elements of a subjective figure, indicates a local processing style. In contrast, attention to the middle/inner part of a subjective figure indicates detection of the illusory contour, that is, extraction of a coherent global structure.

Several theoretical attempts have been made to explain the extraction of illusory contours. For example, the Gestalt view proposes that the contour integration process is based on principles such as good continuation. Alternatively, the pictorial depth cues account states that the inducing elements contain pictorial depth cues that evoke the perception of an illusory contour partially covering the inducing elements.

Much effort has gone into uncovering the neural structures involved in the detection of subjective contours (for reviews, see Murray & Herrmann, 2013; Seghier & Vuilleumier, 2006). Neurophysiological studies established a main contribution of the areas V1 and V2 in the visual cortex (e.g., Larsson et al., 1999; Lee & Nguyen, 2001; Ohtani et al., 2002). From that, some researchers assume that perception of subjective contours is a bottom-up driven process starting within lower cortical levels (e.g., Grosof et al., 1993). The contribution of V1, however, is controversial, and some studies throw doubt on an illusory contour activation in this area (e.g., Ffytche & Zeki, 1996; Peterhans et al., 1989). Alternatively, the top-down approach stresses a predominant influence of higher brain areas on illusory contour formation. Indeed, several studies found an activation by subjective contours in the lateral occipital complex (LOC) (e.g., Mendola et al., 1999; Shpaner et al., 2009; Yoshino et al., 2006). Murray et al. (2002) point out that responsiveness to subjective contours in V1 and V2 may be driven by feedback modulation from the LOC (see also, e.g., Anken et al., 2018; Halgren et al., 2003; Knebel & Murray, 2012; Stanley & Rubin, 2003). In sum, two mechanisms obviously contribute to the perception of subjective contours (Seghier & Vuilleumier, 2006). First, the early visual areas V1 and V2 are engaged in the detection of local features and details. Second, higher visual areas such as the LOC group the features into a global shape. This shape information is then transmitted to V1 and V2 via feedback connections in order to accomplish the boundary completion process.

Csibra et al. (2000) conducted the only neurophysiological study with infants. According to their results, infants 8 but not 6 months of age display adult-like binding-related EEG activity to a subjective Kanizsa square over the left frontal scalp. Most infant studies, however, have used looking methods. One basic finding of these studies is that from about 4 months of age onward infants distinguish between a subjective Kanizsa display and a display with rotated inducing circles (e.g., Bertenthal et al., 1980; Ghim, 1990; Otsuka et al., 2004). For example, according to Ghim (1990), infants 3 and 4 months of age are able to discriminate between a subjective Kanizsa square (Fig. 1a) and a non-illusory Kanizsa figure, the inducing elements of which are rotated by 180° (Fig. 1c). Rotation of the inducing elements deletes the impression of an illusory contour. The difference between the displays is both global and local. The global difference is produced by the presence of the illusory square in the subjective Kanizsa figure and its absence in the non-illusory Kanizsa display. However, instead of responding to the global difference between the displays, the infants might have reacted to their local differences, that is, the orientation differences between the inducing circles. Ghim (1990) therefore conducted a control condition with two non-illusory Kanizsa figures, each of which was created by rotating some of the inducing elements. The difference between these figures is merely local because of the absence of an illusory shape in both. Indeed, the participants did not distinguish between these non-illusory Kanizsa figures. This finding therefore indicates that infants do not to respond to the local differences between two non-illusory Kanizsa displays. From that, Ghim (1990) concluded that infants aged 3 and 4 months are sensitive to the global change between a subjective and a non-illusory Kanizsa figure. Infants not only respond to illusory contours induced by circles with cut-outs but also to illusory contours and illusory lines induced by line endings. Kavšek (2002) established the ability to respond to a subjective Ehrenstein figure with collinear line inducers (see also Fig. 1d) in infants 4 months of age. The experimental procedure employed in that study was similar to that used by Ghim (1990). The non-illusory displays were generated by shifting the inducing lines, that is, by destroying their inner collinearity (see Fig. 1f). Furthermore, infants 2 months of age discern an illusory line generated by aligned line terminators, according to Curran et al. (1999). In a preferential-looking experiment, Bulf et al. (2009) found that infant participants 6 months of age distinguished between a subjective Kanizsa triangle and a non-illusory Kanizsa display. However, unlike adults, the infants failed to detect the subjective Kanizsa figure in a visual search task. From that, it can be concluded that infant responsiveness to subjective figures is not yet adult-like.

The results of several studies with children suggest a protracted evolvement of the binding processes involved in the extraction of illusory contours. Abravanel (1982) asked children aged 3–6 years to describe subjective figures. The identification of illusory shapes could not be reliably determined in the youngest children, increased in particular between 3 and 4 years, and was clearly present in the 6-year-olds. Using eye tracking, Feltner et al. (2010) found that by 6 years of age children primarily inspect the inner part of subjective figures. Children 3–5 years of age, however, concentrate on the inducing elements. In contrast, with real forms, like adults, children of all ages primarily fixate the inner figure part. According to these findings, younger children process subjective figures locally. At 6 years of age, children switch to a global processing mode and become able to extract the illusory contour within subjective figures. In a study conducted by Nayar et al. (2015), adults and children 3–10 years of age were subjected to a match-to-sample task. More specifically, they were shown a real shape and were subsequently asked to pick out from two different illusory Kanizsa contours the one that best suited the real shape. The illusory contours were generated by variations in the number, arrangement, and gap width of the inducing elements. These variations induced, for example, an illusory triangle or an illusory trapezoid. Touching and looking behavior of the participants indicated an age-related gradual increase in the identification of the correct illusory contour. Response accuracy increased between 3 and 8 years of age. By 7–8 years of age, performance was adult-like. In addition, the younger children tended to preferentially look at and touch the inducing elements, whereas the older children tended to predominantly look at and touch the middle part of the subjective figures. Likewise, the adult participants concentrated on the middle part of the subjective figures.

These studies suggest that illusory contour extraction emerges after about 3 years of age. Other studies established adult-like performance in children older than approximately 8 years of age only. Happé (1996) observed that only 57% of the participants 8 years of age were deceived by a subjective Kanizsa triangle. Both Milne and Scope (2008) and Hadad et al. (2010) asked children to discriminate between differently shaped Kanizsa contours. The variations in illusory contouring were created by varying the orientation of the inducing three-quarter circles. Milne and Scope (2008) established a successful discriminatory performance in 60% of their 7- to 11.5-year-old participants. In the Hadad et al. (2010) study, an adult-like performance level was reached by 12 years of age. The discriminatory performance of children aged 6 and 9 years was substantially lower. With real contours, stimulus discrimination did not differ from that of adults by 9 years of age. Hadad et al. (2010) also tested the impact of the support ratio on the perception of Kanizsa figures. The support ratio is defined as the proportion of the length of the real, luminance-defined contour, that is, the length of the contour defined by the inducing elements, to the total length of the sides of the illusory contour, that is, the length of the real and the interpolated components of the illusory shape. The support ratio exerted a strong effect on the perceived strength of illusory Kanizsa contours in adults (see also Altschuler et al., 2012) and in children 9 and 12 years of age. In contrast, perception of illusory contours was independent of the support ratio in children 6 years of age, indicating that contour interpolation is still immature at this age (see also Hadad et al., 2015). Bondarko et al. (2010) asked children 7–17 years of age and adults to describe subjective Kanizsa figures. The ability to perceive illusory Kanizsa squares was substantially lower in children aged 7–12 years than in older children and adults. Altschuler et al. (2014) measured oral and EEG responses to subjective Kanizsa figures in participants 6–31 years of age. Irrespective of age, nearly all participants succeeded in perceiving the illusory contours. The EEG measurements, however, suggested a developmental progress. More specifically, The EEG patterns indicated a prevalence of automatic filling-in processes in the adults. In contrast, these processes were still immature in the participants aged 6–12 years. Instead, these children obviously relied on more effortful processes of comparison of perceived incomplete information with neural object representations. The automatic object completion processes occur in an early stage of contour integration. The automatic processes are complemented by the later effortful filling-in processes that occur when incoming fragmented information is insufficient for object identification (see also, e.g., Murray et al., 2006; Sehatpour et al., 2006; Shpaner et al., 2013). Using eye tracking in a visual search paradigm, Duggan et al. (2019) tested the ability to detect a subjective Kanizsa square amongst arrays with randomly oriented inducing elements in children aged 3–9 years and in adults. Analysis of the participantsʼ saccades to the illusory targets showed that the accuracy of illusory contour detection improved with age in the children, and was highest in the adults. Moreover, visual search efficiency, operationalized by the number of first saccades to the illusory target, in the adults was superior to that in the children. Finally, response accuracy improved with increasing support ratio in both children and adults.

Overall, employing a variety of experimental methods, prior research indicates an age-related increase of the sensitivity to the illusory effects evoked by subjective figures. This finding is consistent with other observations of a transition of visual functions from a local, componential stimulus-processing mode to an integration of local stimulus elements into a global whole. Again, this research can be subdivided into studies in infants and studies in children. In infancy, the transition has been shown for the processing of stimuli containing featural versus configural changes in spatial organization (e.g., Dineen & Meyer, 1980), the detection of angular relations (e.g., Cohen & Younger, 1984), the ability to segregate a figure from the surround (e.g., Sireteanu & Rieth, 1992), the responsiveness to several Gestalt principles (e.g., Quinn et al., 2002; van Giffen & Haith, 1984), and the extraction of featural versus configural information of faces (e.g., Cashon & Cohen, 2004; Schwarzer et al., 2007). In an illustrative habituation-dishabituation study, Quinn et al. (2002) tested infants aged 3–4 and 6–7 months for their ability to group rows and columns of X and O elements according to their form similarity. For example, the infants were habituated to horizontally arranged arrays of X and O elements. During the dishabituation period, they were confronted with a pattern of black horizontal lines versus a pattern of black vertical lines. The older but not the younger infants preferred looking at the vertical lines. That is, unlike the younger age group, the older infants had organized the horizontal arrays of X and O elements into whole horizontal units, had equated them with the black horizontal lines, and had regarded the black vertical lines as novel. To summarize, these studies substantiate that global stimulus perception emerges in the first year of life.

In childhood and adolescence, an age-related improvement of the interpolation of missing stimulus information into a coherent pattern has been reported for the processing of visual hierarchical patterns (e.g., Kimchi et al., 2005; Mondloch et al., 2003; Poirel et al., 2008; Scherf et al., 2009), the use of the principles of proximity and collinearity to integrate unconnected elements into a closed, global form (e.g., Hadad & Kimchi, 2006; Hipp et al., 2014), and the processing of faces in a holistic instead of a part-based manner (e.g., Joseph et al., 2015; Macchi Cassia et al., 2009; Mondloch et al., 2002). For example, Kimchi et al. (2005) asked participants aged 5, 10, 14, and 22 years to judge the similarity of hierarchical arrays that, for example, were squares on the configural level and circles on the local level. Moreover, the configurations were composed of either a few or many elements. On some trials, the similarity judgements had to be made on the basis of the global configuration. On the remaining trials, the similarity judgements had to be made on the basis of the local elements. When the similarity judgements were based on the global configuration, the youngest participants made more errors than the older participants in the few-elements condition. In the many-elements condition, error rates were comparable across age groups. When the similarity classification was based on the local elements, the youngest participants displayed more errors in the many-elements condition. In the few-elements condition, no age differences appeared. Similar age-related changes were established for the speed of classification.

Overall, previous research on the development of illusory contour perception provides a mixed picture. Studies with infants strongly suggest that illusory contour perception emerges in the first year of life (e.g., Bulf et al., 2009; Ghim, 1990). In contrast, studies with children suggest that illusory contour perception emerges after about 3 years or even as late as approximately 8 years of age.

The present study investigated the perception of illusory contours in children aged 3–11 years and adults. The aim of the study was to follow the developmental trajectory of responsiveness to subjective contours in childhood. As even infants have been shown to be sensitive to illusory contours, the study particularly investigated whether or not children aged 3 years extract illusory shape information from subjective figures. Specifically, an eye-tracking technique was employed to establish the areas within subjective figures that were inspected by the participants. Eye tracking has the advantage of being a language-free method, so that even young children can be tested using this paradigm. Indeed, eye tracking has been successfully employed in previous studies on the development of responsiveness to subjective figures in childhood (Duggan et al., 2019; Feltner et al., 2010; Nayar et al., 2015). According to Nayar et al. (2015; see also Feltner et al., 2010), gazing at and touching the inducing elements indicates local processing of the visible component parts, whereas looking at and touching the middle of subjective contours indicates global form perception. This interpretation is validated by the observation of a covariation between performance in the match-to-sample task and looking pattern in children and adults. The authors found that selection of the correct illusory contour and gazing at the middle of the Kanizsa figure instead of the inducing elements increased with age. Neurophysiological evidence corroborates that compared to non-illusory contours, illusory contours activate specific brain areas such as the lateral occipital cortex, even if the participants did not receive any instructions (e.g., Evina et al., 2013; Halgren et al., 2003; Ohtani et al., 2002). Obviously, illusory contours automatically evoke contour-binding processes. Consistent with these observations, visual search studies found that illusory contours embedded in distractors “pop-out”, that is, automatically capture the participants’ visual attention (e.g., Bulf et al., 2009; Conci et al., 2009; Davis & Driver, 1994; Duggan et al., 2019; Gurnsey et al., 1992). Based on these findings, it was hypothesized that the participants would look longer at the visible parts of the subjective stimulus patterns, that is, the inducing elements, if they processed the stimuli in a local, element-based mode. Alternatively, they would look longer at the inner part of the subjective stimuli, that is, the illusory surface, if they processed the stimuli in a global manner.

Unlike earlier studies, the participants were shown not only a subjective Kanizsa figure (Fig. 1a), but also a subjective Ehrenstein figure (Fig. 1d). The experiment could therefore assess whether children and adults process a subjective contour with inducing pitch circles in the same manner as a subjective contour with inducing converging lines. Moreover, the experiment included displays with real contours (Figs. 1b and 1e) to determine whether the looking behavior toward subjective contours equals the looking behavior toward real contours. Psychophysical (e.g., Elliott & Shevell, 2018; Paradiso et al., 1989) and neuropsychological (e.g., Kinsey et al., 2009; Mendola et al., 1999; Pegna et al., 2002) evidence shows that the mechanisms involved in the processing of illusory and real contours overlap. The study therefore also examined whether illusory and real contours evoke comparable looking patterns. Finally, control displays (Figs. 1c and 1f) were included that contained the inducing elements of the subjective patterns but no illusory surface. The looking behavior toward the subjective figures was compared with the looking behavior toward these control displays to elucidate whether looking at the inner and outer parts of the subjective figures was produced by a natural bias toward certain areas of the stimuli. Indeed, several studies established that participants prefer to look at the middle of the scene presented on a computer screen rather than at the outer area (e.g., Parkhurst et al., 2002; Tatler, 2007).

## Method

### Participants

The final sample, consisting of White participants, comprised 21 children aged 3–4 years (12 females, mean age = 4.50 years, range 3.25–4.92 years), 26 children aged 5–6 years (10 females, mean age = 5.94 years, range = 5.08–6.67 years), 27 children aged 7–8 years (15 females, mean age = 7.57 years, range = 7.00–8.25 years), 23 children aged 9–11 years (10 females, mean age = 10.25 years, range = 9.00–11.20 years, and 30 adults (19 females, mean age = 28.17 years, range = 18.75–43.50 years). An additional three children were excluded from the sample because the eye-tracking system did not deliver any data for them. The present study was conducted according to the guidelines laid down in the Declaration of Helsinki. The children’s parents and the adult participants gave written informed consent before any assessment of data collection. The children were recruited in kindergartens and elementary schools, and the adults by word of mouth.

All procedures involving human subjects in this study were approved by the ethics committee of the Department of Psychology at the Rheinische Friedrich-Wilhelms-Universität Bonn, Germany.

### Apparatus

Each participant was seated on a chair in front of a 19-in. flat computer screen. A Tobii X120 eye-tracker was placed between the participant and the computer screen. The center of the screen was at the participant’s eye level. The distance between the participant’s eyes and the eye-tracking device was 60 cm. The distance between the eye tracker and the monitor was 23 cm. The experiment was run using OGAMA software (Vosskühler et al., 2008).

### Stimuli

Each participant was presented with the six stimuli outlined in Fig. 1. Three stimuli depicted variations of the Kanizsa figure: a version with an illusory square (Fig. 1a), a version with a real square (Fig. 1b), and a control display (Fig. 1c). The diameter of the black three-quarter circles was 2.4 cm. In Fig. 1a, the illusory square contour induced by the circles measured 3.68 × 3.68 cm. The real contour shown in Fig. 1b was generated by coloring the area of the illusory square grey. The control display shown in Fig. 1c was generated by rotating the inducing circles in Fig. 1a by 180°. As a result, no illusory contour can be seen in the middle of the stimulus. Similarly, three variations of the Ehrenstein stimulus were constructed: a version with an illusory circle (Fig. 1d), a version with a real circle (Fig. 1e), and a control display (Fig. 1f). The displays consisted of 12 black rectangles 0.62 cm wide. The length of three of the rectangles was 1.44, 1.80, 2.52, and 2.88 cm, respectively. The illusory and the real grey circle in Figs. 1d and 1e had a diameter of 4.52 cm. The control display in Fig. 1f was generated by shifting the inducing rectangles in such a way that their outward pointing ends were arranged in a circle, thereby deleting the percept of an illusory contour in the center of the figure. In the illusory and the real display versions of both the Kanizsa and the Ehrenstein stimulus, the area of the inducing elements was equal to the area of the illusory and the real contour, respectively.

Between trials, an attention-getter displaying a turtle was shown. As soon as the participant looked at the attention-getter, the next trial was initiated.

### Procedure

Each participant was tested individually in a quiet room. The participant was seated comfortably in front of the computer screen. Then, in order to calibrate the eye-tracking device, the participant was asked to fixate each of nine coloured circles, successively presented on different locations across the entire region of the computer screen. If the fixations were not clustered around the calibration circles, calibration was repeated. However, this very rarely occurred. When assessing the calibration data, the experimenters did not establish any differences between the participants. The subsequently collected fixation data were calculated from the calibration values. After the calibration procedure, the participant was presented with the three versions of both the Kanizsa and the Ehrenstein stimulus (see Fig. 1) on successive trials. Each participant was presented with an experimental sequence of three blocks of six trials. In each block of trials, each of the six experimental stimuli was presented once. The Kanizsa and the Ehrenstein stimuli were presented alternately, that is, a Kanizsa stimulus was always followed by an Ehrenstein stimulus and vice versa. Overall 12 blocks of six trials were randomly constructed. These blocks were then divided into four sequences consisting of three blocks each. As a result, each of these experimental sequences comprised 2 (Kanizsa, Ehrenstein) × 3 (illusory display, real display, control display) × 3 (block 1, block 2, block 3) trials. The four sequences of 18 trials were randomly assigned to the participants. The duration of each trial was 6 s.

**Fig. 2:**
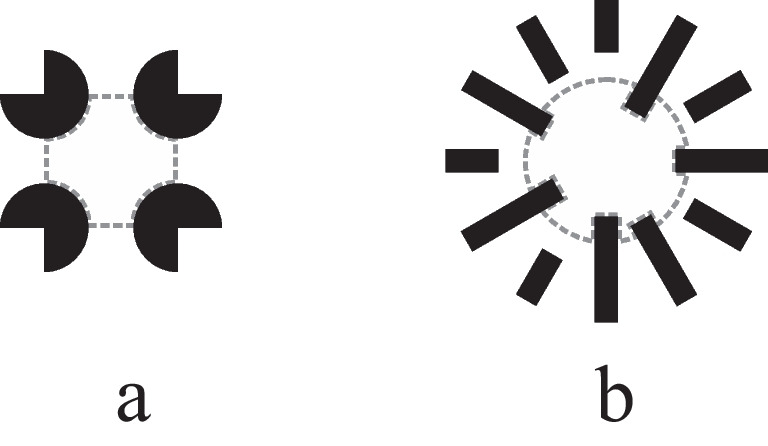
Illustration of the areas of interest (AOIs) of the inner area of the Kanizsa (**a**) and the Ehrenstein (**b**) control display. The AOIs are shown in dashed grey lines

For each stimulus, two areas of interest (AOIs) were determined, the area of the inducing elements and the inner area, that is, the area enclosed by the inducing elements. More specifically, for the illusory and the real displays, the inner stimulus area was the area of the respective contour. For the control displays, the inner area was defined as the area of the real contour (see Figs. 1b and 1e) minus the area within that contour that was occupied by the inducing elements (Fig. 2).

## Results

The dependent variables were extracted from the looking data measured by the eye-tracking device. More specifically, for each stimulus, dwell time, that is, the overall duration the gaze remained in each of the two AOIs, was first determined. These scores were then converted into relative measures. The dwell time toward the inner area of a stimulus was divided by the sum of the dwell time toward the inner stimulus area and the dwell time toward the inducing elements, resulting in a relative dwell time inside score. Relative scores greater than .50 indicate a higher attention to the inner part or rather the global stimulus characteristics of the illusory and the real contour displays. Relative scores less than .50 indicate a higher attention to the inducing elements, that is, a predominantly local processing manner. Overall, data from 2,033 trials were be collected. An additional 253 trials were excluded from data analysis, because no looking to the AOIs was measured. Data loss was highest in the children aged 3–4 years. More specifically, in 25.93% of the measurements, the youngest children did not look at the monitor. The corresponding percentages of the 5- to 6-year-olds, the 7- to 8-year-olds, the 9-to 11-year-olds, and the adults were 11.11%, 3.70%, 4.35%, and 12.41%, respectively. The relatively high loss of measurements in the youngest age group was due to the fact that their attention was easily distracted from the stimuli. They were then motivated by the experimenter to continue the experiment.

The relative dwell time inside scores were analyzed in a 2 × 3 × 5 analysis of variance (ANOVA) with kind of figure (Kanizsa, Ehrenstein), stimulus version (illusory display, real display, control display), and age group (3- to 4-year-olds, 5- to 6-year-olds, 7- to 8-year-olds, 9- to 11-year-olds, and adults) as the independent variables. The analysis yielded a significant (α = .05) main effect of the kind of figure variable, *F*(1, 2003) = 113,21, *p* < .001, *η*_*P*_^*2*^ = .0.05, a significant main effect of stimulus version, *F*(2, 2003) = 119.05, *p* < .001, *η*_*P*_^*2*^ = .11, a significant main effect of age group *F*(4, 2003) = 5.63, *p* < .001, *η*_*P*_^*2*^ = .01, and a significant interaction between kind of figure and stimulus version, *F*(2, 2003) = 6.52, *p* = .002, *η*_*P*_^*2*^ = .01. No other interaction term reached significance. Table 1 contains the combined mean scores of both kinds of figures.

**Table 1:** Combination of the mean relative dwell times inside for the Kanizsa and the Ehrenstein figure for each stimulus version (illusory display, real display, control display) and age group (3- to 4-year-olds, 5- to 6-year-olds, 7- to 8-year-olds, 9- to 11-year-olds, and adults)

	Illusory displays	Real displays	Control displays	All stimulus versions
	*M (SD)*	*M (SD)*	*M (SD)*	*M (SD)*
3- to 4-year-olds	.77 (.35)	.80 (.33)	.58 (.37)	.72 (.36)
5- to 6-year-olds	.84 (.27)	.88 (.24)	.65 (.33)	.79 (.30)
7- to 8-year-olds	.85 (.26)	.92 (.18)	.67 (.32)	.81 (.28)
9- to 11-year-olds	.81 (.28)	.86 (.26)	.64 (.34)	.77 (.31)
Adults	.84 (.27)	.84 (.26)	.63 (.35)	.77 (.31)
All age groups	.83 (.28)	.87 (.25)	.64 (.34)	.78 (.31)

The main effect of kind of figure indicated that the overall mean relative dwell time inside was significantly larger for the Ehrenstein figure, *M* = .84, *SD* = .27, than for the Kanizsa figure, *M* = .71, *SD* = .33.

To analyze the significant effect of the stimulus version factor, Bonferroni-corrected post hoc comparisons between the three stimulus versions were conducted. These tests revealed that the mean relative dwell time inside score for the real displays, *M* = .87, *SD* = .25, was somewhat, but not significantly, greater than that for the illusory displays, *M* = .83, *SD* = .28, *p* = .057. Most importantly, both scores were significantly (both *p*s < .001) greater than the mean relative dwell time inside for the control displays, *M* = .64, *SD* = .34. Furthermore, all means are greater than .50, the chance probability, meaning that the participants looked longer at the inner parts than at the visible outer elements of all experimental displays. The longer looking at specifically the illusory and the real contours cannot be explained as an inherent overall tendency to inspect the inner part of the subjective and the real displays, because the mean relative dwell time inside for the control displays was significantly lower than the mean relative dwell times inside for the illusory and the control displays.

**Fig. 3:**
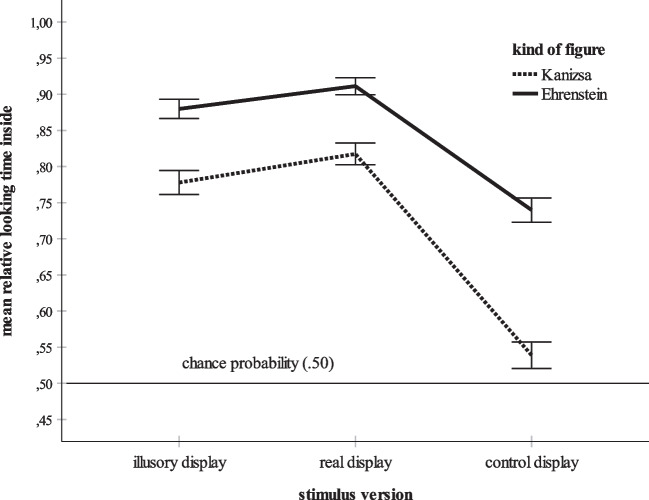
Mean relative dwell time inside for each stimulus version (illusory display, real display, control display) and for each kind of figure (Kanizsa, Ehrenstein). The error bars represent ± 1 *SE*

The main effects of kind of figure and stimulus version are also illustrated in Fig. 3. Figure 3 summarizes the mean looking times inside for each stimulus version of the Kanizsa and the Ehrenstein figure. According to the figure, the significant interaction between kind of figure and stimulus version is obviously due to the large deviation between the mean for the Ehrenstein control display and that for the Kanizsa control display. Bonferroni-corrected simple effects tests were conducted to test the impact of stimulus version (illusory display, real display, control display) on the relative dwell times inside for each kind of figure (Kanizsa, Ehrenstein) and to compare Kanizsa with Ehrenstein for the illusory displays, the real displays, and the control displays separately. The post hoc comparisons revealed that the mean relative dwell time inside scores for the illusory, *M* = .78, *SD* = .31, and the real, *M* = .82, *SD* = .28, Kanizsa displays were significantly greater than the mean score obtained for the control Kanizsa display, *M* = .54, *SD* = .34, both *p*s < .001. The difference between the mean scores for the illusory and the real display was not significant. Similarly, the post hoc analysis of the data for the Ehrenstein figure revealed that there was no significant difference in mean dwell times inside between the illusory display, *M* = .88, *SD* = .24, and the real display, *M* = .91, *SD* = .22. The control display, however, had a significantly lower mean dwell time inside, *M* = .74, *SD* = .31, than the illusory and the real display, both *p*s < .001. In sum, the participants preferred looking at the central area within all Kanizsa and Ehrenstein displays (see also Table 1). Nevertheless, this effect was reliably stronger for the illusory and the real displays than for the control displays. Next, the simple effects analysis compared Kanizsa with Ehrenstein for the illusory displays, the real displays and the control displays separately. For each stimulus version, mean relative dwell time for the Ehrenstein stimulus significantly exceeded the mean relative dwell time for the Kanizsa stimulus, all *p*s < .001.

Bonferroni-corrected *t* tests examined whether the six mean relative looking times depicted in Fig. 3 deviated significantly from the chance probability (.50). The *t* tests revealed that all means, all *p*s < .001, except the mean for the control Kanizsa display, *p* = .069, were significantly greater than the chance score. Hence, with the exception of the control Kanizsa display, the participants inspected the inner parts of the displays significantly longer than their visible outer parts. Table 2 contains the means and the results of the *t* tests.

**Table 2:** Results of the *t* statistics testing the mean relative dwell time inside for each stimulus version (illusory display, real display, control display) of the Kanizsa and the Ehrenstein figure against chance (.50). The *p* scores are two-tailed Bonferroni-corrected *p*s

	*M (SD)*	*t*(2003)	*P*
Illusory Kanizsa display	.78 (.31)	18.16	< .001
Real Kanizsa display	.82 (.28)	20.62	< .001
Control Kanizsa display	.54 (.34)	2.53	.069
Illusory Ehrenstein display	.88 (.24)	24.63	< .001
Real Ehrenstein display	.91 (.22)	26.80	< .001
Control Ehrenstein display	.74 (.31)	15.60	<.001

**Fig. 4:**
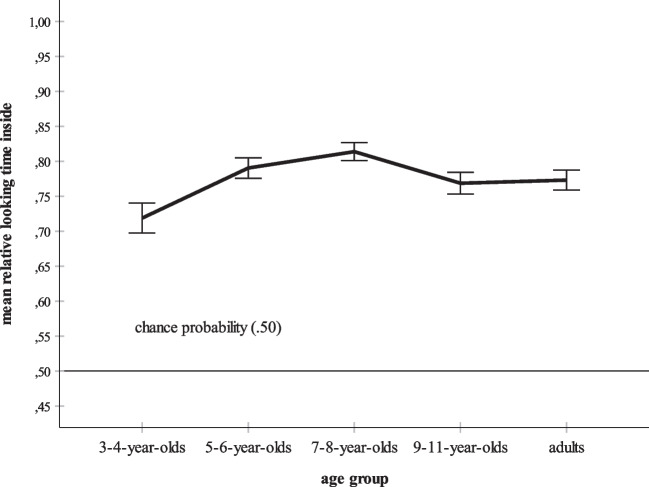
Overall mean relative dwell time inside for each age group (3- to 4-year-olds, 5- to 6-year-olds, 7- to 8-year-olds, 9- to 11-year-olds, and adults). The error bars represent ± 1 *SE*

**Fig. 5:**
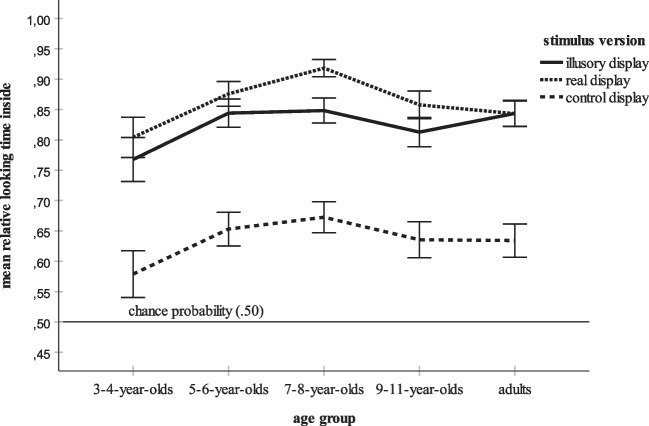
Mean time spent by each age group looking at the inner part of each stimulus version (illusory display, real display, control display). The error bars represent ± 1 *SE*

**Fig. 6:**
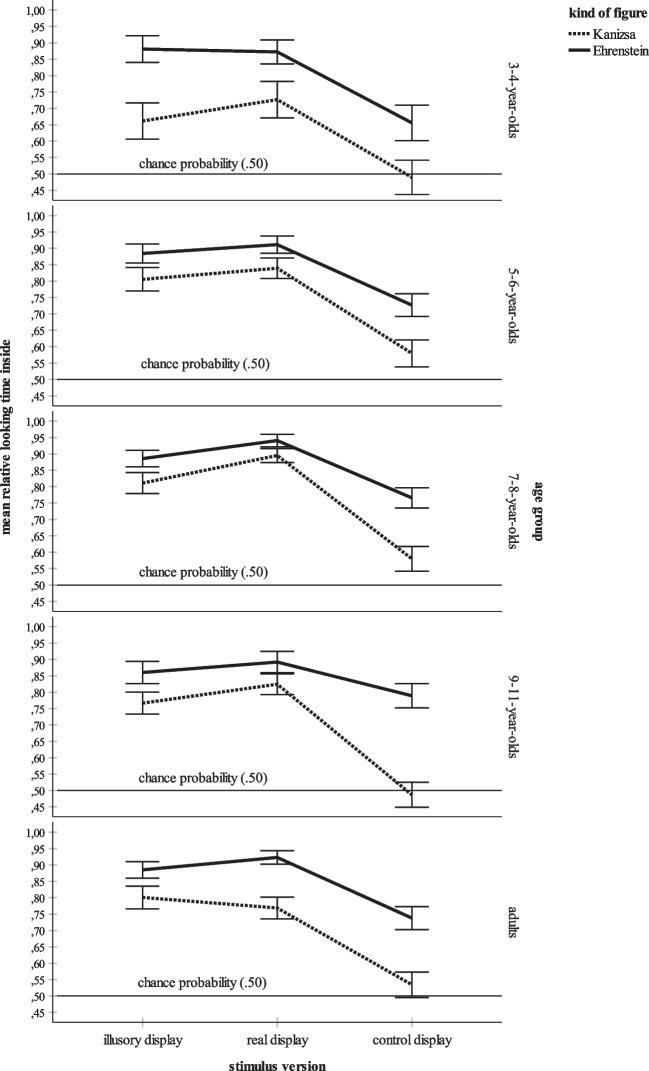
Distribution of the mean relative dwell times inside for each stimulus version (illusory display, real display, control display) and for each kind of figure (Kanizsa, Ehrenstein), separately for the five age groups. The error bars represent ± 1 *SE*

The overall ANOVA also yielded a significant effect of age group. Bonferroni-adjusted post hoc tests found that the overall mean relative dwell time inside score of the youngest participants, the children 3–4 years of age, *M* = .72, *SD* = .36, was significantly lower than the means for the children aged 5–6 years, *M* = .79, *SD* = .30, *p* = .011, and the children aged 7–8 years, *M* = .81, *SD* = .28, *p* < .001. All other post hoc tests failed to reach significance. Figure 4 summarizes the mean relative looking times inside for each age group. For the sake of clarity, Fig. 5 additionally distinguishes between the three stimulus versions. Figures 4 and 5 show an increase of mean looking at the inner part of each stimulus during childhood. At age 5–6 years, the adult-level is reached. For each age group, Fig. 6 additionally elucidates the distribution of the mean looking times for the three versions of both the Kanizsa and the Ehrenstein figure. Figure 6 shows that the main effects of kind of figure and stimulus version and their interaction are basically replicated in each age group. First, the participants within each age group look longer at the interior of the Ehrenstein displays than at that of the Kanizsa displays. Second, the illusory and the real contours evoke longer inspection times than the control contours. Third, in each age group, by far the lowest mean dwell time inside is that for the Kanizsa control display. Finally, all age groups prefer looking at the inner parts of the stimuli, especially of the illusory and real displays.

Bonferroni-corrected *t* tests examined whether the mean relative dwell time inside of each age group exceeded the chance probability score. According to Table 3, all tests were significant, all *p*s < .001. In sum, even though the children aged 3–4 years have the lowest mean relative dwell time inside, they nevertheless inspected the central part of the stimuli significantly longer than the inducing elements, as did all other age groups.

**Table 3:** Results of the *t* statistics testing the overall mean relative dwell time inside for each age group against chance (.50). The *p* scores are two-tailed Bonferroni-corrected *p*s

	*M (SD)*	*t*(2003)	*p*
3- to 4-year-olds	.72 (.36)	12.94	< .001
5- to 6-year-olds	.79 (.30)	20.93	< .001
7- to 8-year-olds	.81 (.28)	23.99	< .001
9- to 11-year-olds	.77 (.31)	18.89	< .001
Adults	.77 (.31)	20.99	< .001

## Discussion

The illusory contour area in both the subjective Kanizsa and subjective Ehrenstein figure attracted the children’s and adults’ attention significantly longer than the figures’ inducing elements. The participants’ looking behavior hence provided evidence to suggest that they filled in the gaps between the inducing elements and extracted the illusory shapes (e.g., Nayar et al., 2015).

The participants were not only shown the illusory Kanizsa and the illusory Ehrenstein pattern but also versions of the figures that contained real contours. Psychophysical and neuroimaging studies describe similarities (e.g., Gegenfurtner et al., 1997; Kinsey et al., 2009; Larsson et al., 1999; Mendola et al., 1999; Vuilleumier et al., 2001) and differences (e.g., Imber et al., 2005; Ramsden et al., 2001) in the perception and the neurophysiological processing of illusory and real contours. For example, speed of contour interpolation is equal for illusory contours and shapes formed by lines, according to Gegenfurter et al. (1997). In contrast, using a backward masking method, Imber et al. (2005) observed that real contours are not as effective as illusory contours. In a functional magnetic resonance imaging study, Mendola et al. (1999) measured brain activity to illusory and real contours. The lateral occipital brain areas activated by illusory contours were the same as those activated by real contours. However, greater activation was obtained for illusory than for real contours. According to the present eye-tracking findings, illusory and real displays evoked highly similar-looking patterns. More specifically, the participants’ mean relative looking time inside toward the illusory contours was not statistically different from their mean relative looking time inside toward the real contours (*p* = .057). The findings are therefore consistent with the view of an overlap of the mechanisms involved in the processing of illusory and real contours. Methodological aspects might determine whether or not an equivalence between illusory and real contours is established. Measuring eye movements is a non-demanding and easy to accomplish task that might therefore be well suited to reveal contour interpolation processes from an early age onward.

Data analysis revealed a significant effect of age group, because the mean relative time spent gazing at the inner parts of all stimuli including the illusory displays increased significantly from age 3–4 years to age 5–8 years (see also Figs. 4,5 and 6). Increases in sensitivity to illusory contours within this age range were also observed by Abravanel (1982), Feltner et al. (2010), and Nayar et al. (2015). However, unlike these studies, the present investigation established clear evidence of illusory contour perception in children 3–4 years of age. Moreover, the results indicate that by 5–6 years of age, subjective contour perception reaches the level of adults. Again, the supposed high sensitivity of eye tracking might explain these differences in findings. Unlike the earlier studies that requested oral descriptions of the stimulus material (Abravanel, 1982) or that used more intricate paradigms such as the combination of looking and touching in a match-to-sample task (Nayar et al., 2015), the present data were collected with a non-verbal, visual measure. Likewise, the use of demanding data collection methods might explain why several studies determined mature extraction of subjective contours in older children only (e.g., Hadad et al., 2015; Milne & Scope, 2008). For example, Milne and Scope (2008) asked their participants to judge whether a Kanizsa contour was either “fat” or “thin.” It might be that the explicit motor and verbal assessment of illusory contours is a task more challenging the processing resources than their mere visual, language-free detection.

In general, earlier psychophysical and neurophysiological research suggests that illusory contours are detected and processed automatically (e.g., Duggan et al., 2019; Evina et al., 2013). Eye tracking is therefore a particularly suitable method to investigate subjective contour perception even in early childhood and in infancy, because it gathers spontaneous visual behavioral data. Moreover, eye tracking allows us to quantify visual behavior. In particular, in the present study, eye tracking was used to explore the distribution of looking over the inducing elements versus the illusory contour area of the Kanizsa and the Ehrenstein figure, indicating a local versus a global processing mode (e.g., Feltner et al., 2010).

The present study is consistent with research on infants. Using looking techniques, many studies provide evidence for infants’ ability to respond to illusory contours (e.g., Otsuka et al., 2004; Sireteanu, 2000; Treiber & Wilcox, 1980). These studies hence substantiate that global processing of subjective figures, that is, illusory contour perception, emerges in the first year of life. To summarize, illusory contours can be distinguished from non-illusory contours and attract the visual attention not only in infancy, but obviously also in early childhood.

Another issue concerns the comparability of the Kanizsa and the Ehrenstein illusions. Mendola et al. (1999) compared brain activity to illusory Kanizsa contours and illusory contours induced by aligned line endings. Both kinds of illusions activated the same lateral occipital brain areas (see also von der Heydt, 2013). The activation evoked by the line endings contours, however, was stronger than that evoked by the Kanizsa contours. Moreover, visual search is more efficient with illusory contours induced by line terminations than with illusory Kanizsa contours (Li et al., 2008). Studies that have dealt with the development of sensitivity to subjective contours have only examined the Kanizsa figure. Our findings therefore confirm and extend the prior research by providing evidence that the illusory effect induced by line endings is indeed stronger than the illusory effect induced by circles with cutouts. Moreover, this superiority of the Ehrenstein over the Kanizsa illusion is not only present in adults but also in children as young as 3–4 years of age.

The control figures were included in the study because several investigations ascertained a pronounced tendency to fixate the center of stimuli on a computer screen more than the periphery (e.g., Tatler, 2007). In the control figures, the subjective surfaces were erased by rotating the three-quarter circles of the Kanizsa figure (Fig. 1c) and by shifts of the lines of the Ehrenstein figure (Fig. 1f). It was hypothesized that the participants would look longer at the inner area of the illusory and the real displays than at the inner area of the control displays if their looking is produced by the perception of the illusory and the real shapes but not by a spontaneous tendency to direct the attention to the inner region of stimuli in general. In fact, the mean relative dwell times inside were substantially greater for the illusory and the real patterns than those for the control patterns. It can therefore be concluded that the participants’ looking behavior was determined by the extraction of illusory and real contour information.

In the illusory and the real contour displays, the size of the inner contour AOI equalled the size of the AOI defined by the inducing elements. In the control displays, however, the inner AOI was smaller than the size of the inducing elements due to the rotation of the inducing circles in the Kanizsa display and the translations of the inducing lines in the Ehrenstein display. As a result, the size of the inner AOI was smaller in the control displays compared to the size of the inner AOIs in the illusory and the real displays (see Fig. 2). As a consequence, if there had been a spontaneous tendency to preferentially look at the inner stimulus part, it might have been somewhat attenuated in the control displays relative to the subjective and real displays. The use of the control displays as controls of natural preferences for the inner figure part should be interpreted with these reservations in mind.

The present study exploited eye movements directed at the different parts of illusory, real, and control displays. Eye tracking is an unobtrusive tool for automated tracking of gaze behavior. Using this method, the study established that even children aged 3–4 years respond to illusory contours. Moreover, illusory contour perception obviously improves during the preschool period and becomes adult-like at the age of 5–6 years. Future developmental research should test a wider age range and combine different methods. In particular, infants and children younger than 3 years of age should be included to more closely follow the development of subjective contour perception. Moreover, looking data should be enriched with neurophysiological measures to reveal the developmental trajectory of the underlying brain mechanisms.

## Data Availability

The data generated and analyzed during the current study and scripts for performing data analysis will be freely available from the corresponding author on reasonable request. The study was not preregistered.
